# The association of dietary glutamine supplementation with the development of high salt-induced hypertension in rats

**DOI:** 10.3389/fnut.2022.1011739

**Published:** 2022-11-15

**Authors:** Liu Yang, Longjin Xu, Juan Li, Huan Wang, Jiahong Sun, Ziqiang Yu, Xiaoqian Zhao, Min Zhao, Bo Xi

**Affiliations:** ^1^Department of Epidemiology, School of Public Health, Cheeloo College of Medicine, Shandong University, Jinan, China; ^2^Centers for Disease Control and Prevention of Shandong Province, Jinan, China; ^3^Department of Nutrition and Food Hygiene, School of Public Health, Cheeloo College of Medicine, Shandong University, Jinan, China

**Keywords:** L-glutamine, blood pressure, hypertension, high salt, rates

## Abstract

Glutamine supplementation has been reported to affect blood pressure (BP). However, its role in the progression of hypertension induced by high salt diet (HSD) has not been elucidated. Male normotensive Wistar rats were exposed to high salt diet and treated with different doses of glutamine supplementation. Rats aged 6 weeks were assigned to five groups: (1) Normal-salt diet (0.3% NaCl, NSD); (2) High-salt diet (8% NaCl, HSD); (3) High-salt + low-dose diet (8% NaCl, 0.5 g of L-glutamine/kg body weight, HSLGD); (4) High-salt + middle-dose diet (8% NaCl, 1.5 g of L-glutamine/kg body weight, HSMGD); and (5) High-salt + high-dose diet (8% NaCl, 2.5 g of L-glutamine/kg body weight, HSHGD). After supplementing different doses of glutamine to male Wistar 6-week-old rats fed with HSD for 7 weeks, we found no difference in body weight among groups. Importantly, we showed that dietary L-glutamine supplementation could prevent the development of hypertension in a dose-dependent manner [dramatically lowering systolic blood pressure (SBP) and slightly reducing diastolic blood pressure (DBP) of hypertensive rats, while the differences of DBP between groups did not reach statistical significance]. Our data further elucidated that dietary glutamine supplementation mildly alleviated the degree of left ventricular hypertrophy, including interventricular septal thickness (IVST) and left ventricular posterior wall thickness (LVPWT) in hypertensive rats. Together, our results offer evidence that the dietary uptake of glutamine may be associated with attenuating the development of high salt-induced hypertension and slightly alleviating the degree of left ventricular hypertrophy in hypertensive rats. Therefore, glutamine supplementation may act as a prospective dietary intervention for the treatment of hypertension.

## Introduction

It is well established that hypertension, one of the most common chronic diseases, is the leading risk factor for heart attack, stroke, congestive heart failure, and kidney disease (1). Hypertension and its complications globally account for 9.4 million deaths among the 17 million deaths owing to cardiovascular diseases each year, and thus has become one of the most serious public health issues across the world (2, 3). Accumulating evidence indicates high dietary salt to be an independent risk factor for chronic non-communicable diseases, especially hypertension, thereby triggering more than half of diet-related deaths around the world (4–6). Therefore, it is urgent to seek for effective dietary intervention strategies to alleviate the occurrence and development of hypertension caused by a high salt diet (HSD).

Previous work has demonstrated that, compared with individuals who had normal blood pressure (BP), patients with hypertension often have concurrent metabolic abnormalities, mainly affecting the metabolism of amino acids, fatty acids, carbohydrates, and the intestinal microbiota (7–11). Glutamine, the most plentiful free amino acid in human serum, attributes to cell survival and growth in a similar fashion to glucose (12). Glutamine is the major nitrogen source for non-essential amino acids, hexosamines, and nucleotides (13) and further plays a role in providing intermediates (like α-ketoglutarate) to the tricarboxylic acid cycle. Liu et al. (10) reported that the level of glutamine was increased in hypertensive patients by the use of ultrasonication-assisted extraction and derivatization. Conversely, another metabolomics study showed that systolic blood pressure (SBP) and pulse pressure are inversely correlated with glutamine in black adults (14). Similarly, our previous work identified that in children aged 6−11 years with elevated BP, the abundance of glutamine was lower than those with normal BP using a case-control design (15). L-citrulline, the metabolite of glutamine, can increase the synthesis of NO and inhibit arterial tension, which may be the way to regulate BP (16). However, the conflicting data means that it remains elusive whether or not glutamine is involved in the development of hypertension.

Therefore, in this experimental study, we treated those high-salt diet-induced hypertensive rats with three different doses of dietary glutamine to preliminary explore the relationship involved between long-term glutamine intake and hypertension induced by HSD in Wistar rats. In addition, we further and firstly evaluated the effect of glutamine intake on the cardiovascular structure [including carotid intima-media thickness (cIMT) and left ventricular hypertrophy] in rats.

## Materials and methods

### Animals and treatment

A total of 65 Wistar 4-week-old rats (140.2 ± 8.8 g) were obtained from Beijing Vital River Laboratory Animal Technology Co., Ltd. All rats were male, considering that the prevalence of hypertension is higher in men than in women (17). One rat was housed per cage in 370 × 260 × 170 mm^3^ cages at constant temperature (18−24°C), humidity (45%), and regular 12 h light/dark cycles with lights on from 06:30 to 18:30 (light = 270 lux). Animals had free access to water and food, except where noted. All animal experimental procedures were approved by the Ethics Committee of the School of Public Health, Shandong University (No. 20160308) and conformed to the Helsinki Declaration.

After 14 days of adaptive feeding, male normotensive Wistar rats aged 6 weeks were randomized to five groups according to BP values and body weight (*n* = 13 per group): (1) normal-salt diet group (a standard normal diet with 0.3% NaCl, NSD); (2) high-salt diet group (a high-salt diet with 8% NaCl, HSD); (3) high-salt + low glutamine diet group (a high-salt diet with 0.5 g of L-glutamine/kg body weight, HSLGD); (4) high-salt + middle glutamine diet group (a high-salt diet with 1.5 g of L-glutamine/kg body weight, HSMGD); and (5) high-salt + high glutamine diet group (a high-salt diet with 2.5 g of L-glutamine/kg body weight, HSHGD) (18). All standard normal diets and high-salt diets were customized by Jiangsu Xietong Pharmaceutical Bio-engineering Co., Ltd. They were all identical in composition except for NaCl content (control: 0.3% NaCl, hypertension: 8% NaCl) (19) and irradiated by Co-60 to meet SPF level. After fully mixing the glutamine and standard high-salt feed using the proportional multiplication method (HSHGD: 33.3 g L-glutamine/1 kg high-salt feed; HSMGD: 20 g L-glutamine/1 kg high-salt diet; HSLGD: 6.67 g L-glutamine/1 kg high-salt diet), three different doses of high-salt feed containing three different doses of glutamine were remade. Oven drying (40°C) and UV sterilization (duration was set to 3 h) were sequentially carried out after feed production. Body weights were monitored every 3 days and pre-weighted food was administered accordingly. After 7 weeks of dietary treatment followed by a 12 h fast, all rats were sacrificed.

### Blood pressure measurements of conscious rats and definitions

Blood pressure measurements were performed from 8:00 a.m. to 6:00 p.m. in a warm and quiet room once a week. A rat was selected randomly for BP measurement from group A, followed by B, C, D, and E, sequentially. After one round, repeat the abovementioned operations until all BP measurements are completed. Systolic blood pressure and diastolic blood pressure (DBP) of conscious rats were measured by the tail-cuff system (BP-2010A, Softron, China) as described previously (19). Five BP measurements were recorded consecutively for each rat. If the difference between any two of the five BP readings per individual rat exceeded 10 mmHg, a sixth BP measurement was conducted. The mean value was calculated for analysis. Hypertension was defined as a SBP ≥ 140 mmHg.

### Ultrasound imaging of the carotid artery and left ventricular function

After 7 weeks of dietary treatment, six rats per group were randomly chosen to be anesthetized using isoflurane (mixed in 95% oxygen and 5% carbon dioxide oxygen) for ultrasonic measurement using small animal color Doppler ultrasound device (Vevo 2100, Visual sonics, Canada). Skin preparation was conducted from the mandible to the upper abdomen and depilatory cream (Nair™) was applied. Rats were positioned on a heated platform in supine position. For measurements of both echocardiogram and ultrasound vessel internal diameter imaging, a trained user of the Vevo 2100 Imaging system, blinded to the intervention of all rats (intervention and dose), analyzed heart and vessel diameters of each rat. All images were saved as cine loops for the subsequent measurement and analyses of vessel and cardiac parameters.

Ultrasound gel was applied to prepared skin. The transducer head was locked (40 MHz probe; MS550D) in the adjustable arm of the Vevo mechanical rail-system to allow hands-free precise positioning of the transducer during cIMT image collection in M-mode. The anterior and posterior walls of cIMT of the right and left were measured, and the mean cIMT was calculated for statistical analysis. Another transducer head (15 MHz probe; MS201) was locked to detect the structure of left ventricle. The parameters included left ventricular end-systolic diameter (LVSD), left ventricular end-diastolic diameter (LVDD), interventricular septum thickness (IVST), and left ventricular posterior wall thickness (LVPWT) by performing M-mode echocardiography. The left ventricular mass (LVM) was calculated according to the Devereux formula: LVM (g) = 0.8{1.04[(LVDD + IVST + LVPWT)^3^- LVDD^3^]} + 0.6 (20).

### Statistical analysis

All data were presented as means ± SEM. Statistical analyses and Graphs were performed using Graphpad Prism software (v.7) (GraphPad Software, La Jolla, CA, United States). For the comparison of more than two groups, one-way ANOVA followed by Bonferroni’s *post-hoc* test was used to determine the statistical significance of differences. A *P*-value less than 0.05 was considered to be statistically significant.

## Results

### Glutamine supplementation had no effect on body weight

**FIGURE 1:**
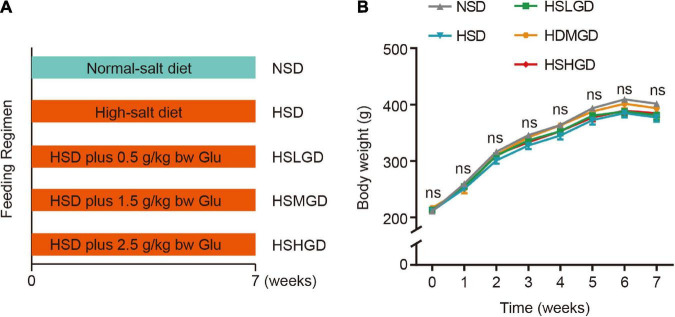
Effects of dietary glutamine on body weight in salt-induced hypertensive rats. **(A)** Schematic outlines of the feeding regimen for the five intervention groups. Male normotensive Wistar rats aged 6 weeks were assigned to five groups: (1) Normal-salt diet (0.3% NaCl, NSD); (2) High-salt diet (8% NaCl, HSD); (3) High-salt + low-dose diet (8% NaCl, 0.5 g of L-glutamine/kg body weight, HSLGD); (4) High-salt + middle-dose diet (8% NaCl, 1.5 g of L-glutamine/kg body weight, HSMGD); (5) High-salt + high-dose diet (8% NaCl, 2.5 g of L-glutamine/kg body weight, HSHGD). **(B)** Body weight of rats fed on NSD, HSD, HSLGD, HSMGD, or HSHGD for 7 weeks. *n* = 12 (NSD, HSLGD) or 13 (HSD, HSMGD, HSHGD) rats per group. Data are presented as means ± SEM. ns, no significance. One-way ANOVA followed by Bonferroni’s *post-hoc* test **(B)**.

The dietary intervention program and grouping of rats are displayed in Figure 1A. We supplemented different doses of glutamine to male Wistar 6-week-old rats fed with HSD for 7 weeks. We did not observe any significant difference in body weight among the five groups (Figure 1B and Supplementary Table 1), indicating that glutamine supplementation in diet did not induce significant changes in the body weight of HSD-induced rats.

### Dietary glutamine prevented high salt diet-induced increases in blood pressure in rats

**FIGURE 2:**
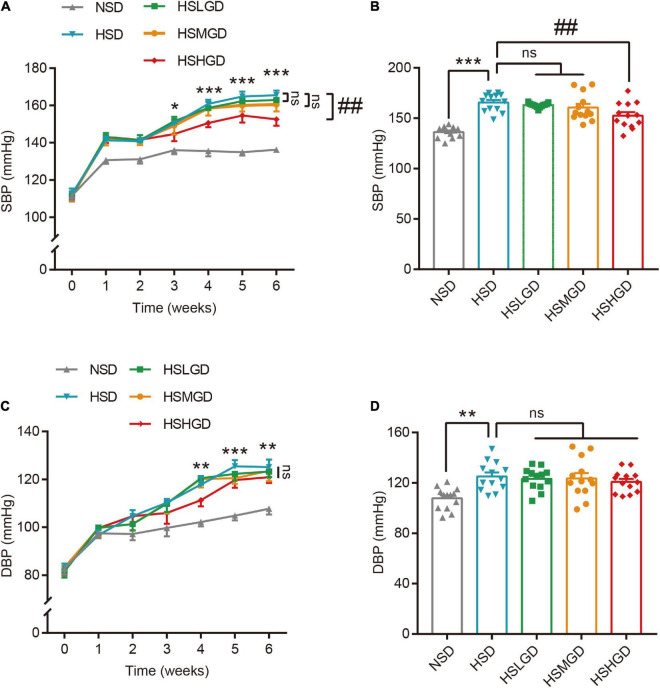
Effects of dietary glutamine on blood pressure (BP) in salt-induced hypertensive rats. **(A,B)** Rats were fed on a normal-salt diet (NSD), high-salt diet (HSD), high-salt + low-dose glutamine diet (HSLGD), high-salt + middle-dose glutamine diet (HSMGD), or high-salt + high-dose glutamine diet (HSHGD) for 7 weeks. Systolic blood pressure (SBP) was measured every week by the tail-cuff system. The changes of SBP from 1 to 6 weeks **(A)** and at 6-week feeding intervention **(B)**. **(C,D)** The diastolic blood pressure (DBP) of rats fed on NSD, HSD, HSLGD, HSMGD, or HSHGD from 1 to 6 weeks **(C)** and at 6-weeks of feeding intervention **(D)**. *n* = 13 rats per group. Data are presented as means ± SEM. *P < 0.05, **P < 0.01, ***P < 0.001, compared with NSD group; ##*P* < 0.01 compared with HSD group. ns: no significance. One-way ANOVA followed by Bonferroni’s *post-hoc* test **(A–D)**.

Blood pressure measurement data showed 6-week HSD increased both the SBP and DBP levels in rats. Intriguingly, we found that 6-week of high-dose dietary glutamine supplementation dramatically prevented the increase of SBP (152.6 mm Hg) (Figures 2A,B and Supplementary Table 2A) and slightly prevented of DBP (120.9 mm Hg) (Figures 2C,D and Supplementary Table 2B) in hypertensive rats compared with HSD-fed rats (SBP: 165.6 mm Hg, DBP: 125.1 mm Hg), while there was not obvious difference of BP for low- and middle-dose dietary glutamine supplementation.

### High glutamine delayed the development of left ventricular hypertrophy in hypertensive rats

**FIGURE 3:**
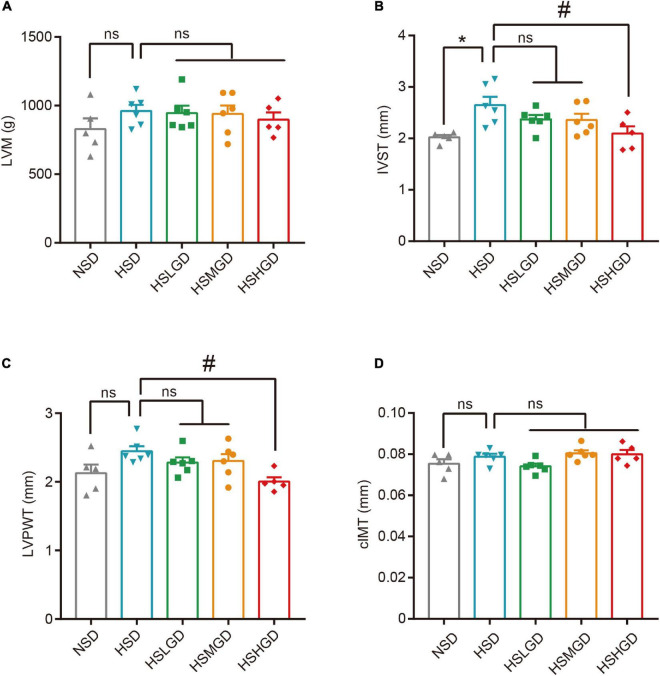
Effects of dietary glutamine on the carotid artery and heart in salt-induced hypertensive rats. **(A–D)** Rats were fed on a normal-salt diet (NSD), high-salt diet (HSD), high-salt + low-dose glutamine diet (HSLGD), high-salt + middle-dose glutamine diet (HSMGD), or high-salt + high-dose glutamine diet (HSHGD) for the 7-week rearing experiment. Data exhibit the indicated ultrasound parameters including LVM **(A)**, IVST **(B)**, LVPWT **(C)**, and cIMT **(D)** measured at 7 weeks. *n* = 5 (NSD, HSHGD) or 6 (HSD, HSLGD, HSMGD) rats per group. Data are presented as means ± SEM. *P < 0.05 compared with NSD group; #*P* < 0.05 compared with HSD group. ns, no significance. One-way ANOVA followed by Bonferroni’s *post-hoc* test **(A–D)**. LVM, left ventricular mass; IVST, interventricular septal thickness; LVPWT, left ventricular posterior wall thickness; cIMT, carotid intima-media thickness.

**FIGURE 4:**
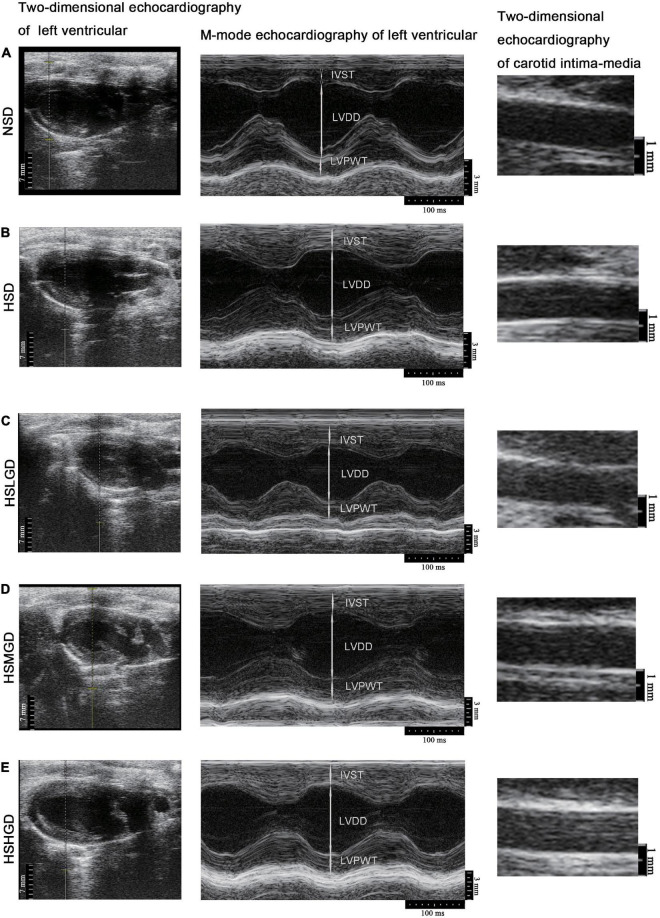
Representative echocardiography images of carotid arteries and cardiovasculature of rats fed on a normal-salt diet (NSD, **A**), high-salt diet (HSD, **B**), high-salt + low-dose glutamine diet (HSLGD, **C**), high-salt + middle-dose glutamine diet (HSMGD, **D**), or high-salt + high-dose glutamine diet (HSHGD, **E**) for the 7-week rearing experiment. Scale bars, 7 mm (Two-dimensional echocardiography of left ventricular), 3 mm (M-mode echocardiography of left ventricle), or 1 mm (Two-dimensional echocardiography of carotid intima-media). *n* = 5 (NSD, HSHGD) or 6 (HSD, HSLGD, HSMGD) rats per group.

We further sought to elucidate the role of a glutamine-rich diet on the function of both the carotid artery and left ventricle in high salt-induced hypertensive rats. Given that ultrasound imaging is a crucial tool for the assessment of the carotid artery and left ventricle structure, we performed ultrasound measurements of the carotid arteries and left ventricles after 7 weeks of feeding treatment. The data indicated that the IVST level was elevated in HSD-fed rats (2.649 mm), while supplemental high-dose glutamine markedly diminished this effect (2.095 mm) (Figure 3B and Supplementary Table 3). Similar results were observed on LVPWT (2.447 mm vs. 2.004 mm) (Figure 3C and Supplementary Table 3). However, no difference in LVM and carotid IMTs was found among different groups (Figures 3A,D and Supplementary Table 3). Two-dimensional echocardiography and M-mode echocardiography revealed that the thickness of the left ventricular wall and carotid intima-media were decreased in glutamine-supplemented groups (HSLGD, HSMGD, and HSHGD) compared with the HSD group (Figure 4).

## Discussion

Accumulating evidence indicates that glutamine plays a crucial role in multiple physiological and pathological statuses. Here, we used an *in vivo* model to explore the role of dietary glutamine supplementation in the development of hypertension induced by HSD in Wistar rats. We found that glutamine supplementation might be inversely associated with elevated SBP and left ventricular hypertrophy in the hypertensive rat model. Our work highlights the pivotal *in vivo* effect of glutamine supplementation on the progression of salt-induced hypertension, paving the way for better therapeutic strategies.

Glutamine serves as an essential nutrient for the synthesis of a number of important molecules, including lipids, proteins, and DNA, and provides intermediates for the tricarboxylic acid cycle to generate ATP. Recently, the role of glutamine in the cardiovascular system has drawn considerable attention (21–23). Glutamine-cycling pathways might be prominently involved in the development of metabolic disorders. Prior studies have observed that serum glutamine levels were inversely linked to the development of obesity and other established risk factors for cardiometabolic disease (24–26). The inversely association of glutamine with metabolic disorders might be due to pancreatic β-cell insulin secretion, increased insulin sensitivity of adipose tissue, transcription of insulin-dependent enzymes, enhanced release of glucagon-like peptide 1, and externalization of glucose transporter type 4 (27–29). Cheng et al. (30) also found that glutamine was inversely related to insulin levels, SBP, and DBP, and positively associated with high-density lipoprotein levels based on two large community cohorts (the Malmo Diet and Cancer Study and the Framingham Heart Study). They further interrogated the regulation of administered glutamine on BP in C57BL/6 mice and found that SBP, DBP, and mean arterial pressure were significantly lower in glutamine-treated mice (intraperitoneal injection of glutamine plus saline) compared with controls (injection of saline alone), which is consistent with our results. Glutamine generates L-citrulline, which is metabolized to L-arginine (nitric oxide synthase substrate) in the kidney through the synergistic action of arginosuccinate synthetase and arginine succinate lyase (31). The suppressive effect of glutamine on BP may be partly attributable to its role as a precursor of L-arginine, leading to the increase of NO synthesis (16), which regulates BP by the inhibition of arterial tone. It is noteworthy that in this animal study by Cheng et al. (30), the BP measurements were conducted every 12 min for 36 min following intraperitoneal injection of glutamine plus saline or saline alone, which reflected transient BP changes in response to the acute administration of glutamine. However, we observed a long-term antihypertensive effect of glutamine on HSD-induced hypertensive rats after a 7-week dietary glutamine intervention.

Current studies have identified the mitigation effects of glutamine on myocardial dysfunction. Clinical trials have observed that glutamine supplementation decreased the post-operative myocardial damage after coronary revascularization in cardiopulmonary bypass (32), increased the concentration of troponin at 24 h after operation, and improved myocardial function for patients with ischemic heart disease (33). In addition, glutamine administration improved contractile function of the left ventricle and protected from cardiac injury in diabetic rats induced by streptozotocin-nicotinamide, which acts through significantly reducing the levels of cardiac enzymes such as creatine kinase-isoenezyme, lactate dehydrogenase, and aspartate aminotransferase (34). Similar protective effects of glutamine on myocardial structure and function have also been found in severely burned rats (35). Our current work highlights that a high glutamine diet may exert alleviative effects on cardiac structural changes caused by HSD-induced hypertension in Wistar rat models. Further work is needed to unveil the mechanisms involving glutamine in the progression of myocardial dysfunction caused by hypertension.

This study also preliminarily explored the relationship between glutamine and carotid artery intima-media thickness. However, no difference in carotid IMTs was found among different groups. Addabbo et al. found that glutamine supplementation corrected endothelium-dependent relaxation in mice treated with L-Nω-methylarginine (36). Similarly, other experimental studies observed that glutamine guarded endothelial cells against inflammation and oxidative stress (37, 38). The protective mechanism of glutamine on blood vessels still requires investigation.

There were certain limitations should be acknowledged. First, considering the water solubility of glutamine, the way of supplementing glutamine is oral rather than gavage, which might lead to partial glutamine loss. Second, we measured only a limited indexes to represent the status of rat’s hypertension to conduct a preliminary explore of the relationship between long-term glutamine intake and hypertension induced by HSD in Wistar rats. Further studies are needed to better understand the mechanisms underlying the effect of dietary glutamine supplementation on HSD-induced hypertension.

## Conclusion

In conclusion, our study revealed that a high glutamine diet might be inversely associated with the development of hypertension and left ventricular hypertrophy in HSD-induced hypertensive rats in a dose-dependent manner. Our work provides pivotal evidence that dietary glutamine supplementation may be used as a promising therapeutic tactic for the treatment of hypertension. The underlying molecular mechanisms of dietary glutamine supplementation on HSD-induced hypertension are worth further exploration both *in vivo* and *in vitro*.

## Data availability statement

The original contributions presented in the study are included in the article/Supplementary material, further inquiries can be directed to the corresponding authors.

## Ethics statement

The animal study was reviewed and approved by Ethics Committee of the School of Public Health, Shandong University. Written informed consent was obtained from the owners for the participation of their animals in this study.

## Author contributions

LY and LX conducted the animal experiment, collected the data, and drafted the manuscript. JL analyzed the data, prepared the figures, and co-wrote the manuscript. HW and ZY assisted in animal experiments. XZ assisted in ultrasound information acquisition and reading. HW, JS, ZY, XZ, MZ, and BX critically revised the manuscript for important intellectual content. BX was the guarantor. MZ and BX conceived the study, designed the experiments, analyzed the data, co-wrote the manuscript, and attested that all the listed authors meet the authorship criteria and that no others meeting the criteria have been omitted. All authors have read and agreed with the final version of this manuscript.
