# Supplementary material for: The association of dietary glutamine supplementation with the development of high salt-induced hypertension in rats
Source: Front Nutr. 2022 Nov 15;9:1011739. doi: 10.3389/fnut.2022.1011739 (PMC9706238; doi:10.3389/fnut.2022.1011739)
Supplement: Supplementary file 1 [file Data_Sheet_1.pdf]

Table S1 Body weight (g) of rats fed on NSD, HSD, HSLGD, HSMGD, or HSHGD for 7 weeks.

	Group	Time (weeks)							
		0	1	2	3	4	5	6	7
**Mean±SEM**	NSD	211.06±3.01	259.47±3.12	316.33±3.42	345.77±3.78	363.54±5.56	393.63±5.02	409.33±5.33	401.55±5.02
	HSD	211.69±3.17	250.65±3.72	300.97±5.82	327.53±6.52	345.28±7.23	372.20±7.88	384.95±7.77	377.07±7.76
	HSLGD	213.17±4.01	254.60±4.68	309.29±6.76	336.61±7.10	352.73±7.55	380.20±7.55	387.62±8.65	381.42±8.38
	HSMGD	216.72±3.17	250.78±8.30	311.22±7.00	341.88±6.64	362.93±7.14	387.83±7.73	401.55±8.44	393.55±8.35
	HSHGD	211.08±2.77	251.78±2.94	311.32±3.88	333.23±4.76	352.77±5.57	377.19±6.74	388.99±8.19	384.39±6.81
***P* value**	HSD vs. NSD	>0.999	>0.999	0.580	0.342	0.591	0.382	0.322	0.236
	HSLGD vs. HSD	>0.999	>0.999	>0.999	>0.999	>0.999	>0.999	>0.9999	>0.99
	HSMGD vs. HSD	>0.999	>0.999	>0.999	0.867	0.627	>0.999	>0.9999	>0.999
	HSHGD vs. HSD	>0.999	>0.999	>0.999	>0.999	>0.999	>0.999	>0.9999	>0.999

Table S2A The changes of systolic blood pressure (SBP, mm Hg) of rats fed on NSD, HSD, HSLGD, HSMGD, or HSHGD for 6 weeks

	Group	Time (weeks)						
		**0**	**1**	**2**	**3**	**4**	**5**	**6**
**Mean±SEM**	NSD	111.60±2.53	130.71±2.02	131.17±2.34	136.00±2.06	135.54±2.83	134.88±1.88	136.29±1.52
	HSD	112.52±2.90	141.32±2.05	140.74±1.85	150.28±1.11	160.86±2.22	164.85±2.67	165.57±2.44
	HSLGD	111.54±2.37	143.03±2.24	141.34±2.84	151.58±2.31	158.71±3.31	162.34±1.99	162.94±0.63
	HSMGD	111.69±3.28	141.98±3.51	141.49±2.72	149.22±4.84	158.63±4.03	160.05±3.31	160.52±3.71
	HSHGD	111.55±2.17	143.14±3.25	141.58±2.84	144.66±3.81	150.57±2.25	154.66±3.84	152.60±3.48
***P* value**	HSD vs. NSD	>0.999	0.071	0.101	0.020*	<0.001***	<0.001***	<0.001***
	HSLGD vs. HSD	>0.999	>0.999	>0.999	>0.999	>0.999	>0.999	>0.999
	HSMGD vs. HSD	>0.999	>0.999	>0.999	>0.999	>0.999	>0.999	>0.999
	HSHGD vs. HSD	>0.999	>0.999	>0.999	>0.999	0.185	0.138	$0.009^{\#\#}$

Table S2B The changes of diastolic blood pressure (DBP, mm Hg) of rats fed on NSD, HSD, HSLGD, HSMGD, or HSHGD for 6 weeks

	**Group**	**Time (weeks)**						
		**0**	**1**	**2**	**3**	**4**	**5**	**6**
**Mean±SEM**	NSD	82.82±2.55	97.45±1.96	97.23±2.61	99.75±3.50	102.18±1.87	104.85±1.96	107.74±2.42
	HSD	82.91±1.95	96.78±2.01	104.63±2.55	110.28±1.59	117.82±3.14	125.43±2.62	125.12±3.15
	HSLGD	81.57±2.62	99.88±3.06	101.32±2.77	109.82±3.49	120.51±2.75	122.29±2.82	123.31±2.39
	HSMGD	83.35±2.37	99.71±3.64	101.38±2.53	110.05±4.28	120.12±3.53	120.46±2.42	123.57±4.26
	HSHGD	81.68±1.76	99.65±2.71	104.62±3.30	106.00±4.48	111.32±2.62	119.72±3.23	120.89±2.40
***P* value**	HSD vs. NSD	>0.999	>0.999	0.635	0.439	0.003**	<0.001***	0.001**
	HSLGD vs. HSD	>0.999	>0.999	>0.999	>0.999	>0.999	>0.999	>0.999
	HSMGD vs. HSD	>0.999	>0.999	>0.999	>0.999	>0.999	>0.999	>0.999
	HSHGD vs. HSD	>0.999	>0.999	>0.999	>0.999	>0.999	>0.999	>0.999

Table S3 Effects of dietary glutamine on the carotid artery and heart in salt-induced hypertensive rats

	Group	LVM (g)	IVST (mm)	LVPWT (mm)	cIMT (mm)
**Mean±SEM**	NSD	829.395±77.224	2.021±0.045	2.125±0.127	0.075±0.002
	HSD	961.089±44.981	2.649±0.158	2.447±0.072	0.079±0.001
	HSLGD	946.051±53.941	2.369±0.085	2.282±0.073	0.074±0.001
	HSMGD	938.865±62.029	2.359±0.120	2.303±0.101	0.081±0.001
	HSHGD	898.245±52.062	2.095±0.139	2.004±0.062	0.080±0.002
***P* value**	HSD vs. NSD	>0.999	0.012*	0.193	>0.999
	HSLGD vs. HSD	>0.999	0.986	>0.999	0.468
	HSMGD vs. HSD	>0.999	0.880	>0.999	>0.999
	HSHGD vs. HSD	>0.999	0.035#	0.021#	>0.999
